# A Cerebellar Neuroprosthetic System: Computational Architecture and *in vivo* Test

**DOI:** 10.3389/fbioe.2014.00014

**Published:** 2014-05-21

**Authors:** Ivan Herreros, Andrea Giovannucci, Aryeh H. Taub, Roni Hogri, Ari Magal, Sim Bamford, Robert Prueckl, Paul F. M. J. Verschure

**Affiliations:** ^1^Synthetic Perceptive, Emotive and Cognitive Systems group (SPECS), Universitat Pompeu Fabra, Barcelona, Spain; ^2^Psychobiology Research Unit, Tel Aviv University, Tel Aviv, Israel; ^3^Physics Laboratory, Istituto Superiore di Sanità, Rome, Italy; ^4^Guger Technologies OG, Graz, Austria; ^5^Institució Catalana de Recerca i Estudis Avançats, Barcelona, Spain

**Keywords:** neuroprosthetics, cerebellum, classical conditioning, timing, memory, association learning, nucleo-olivary pathway, inferior olive

## Abstract

Emulating the input–output functions performed by a brain structure opens the possibility for developing neuroprosthetic systems that replace damaged neuronal circuits. Here, we demonstrate the feasibility of this approach by replacing the cerebellar circuit responsible for the acquisition and extinction of motor memories. Specifically, we show that a rat can undergo acquisition, retention, and extinction of the eye-blink reflex even though the biological circuit responsible for this task has been chemically inactivated via anesthesia. This is achieved by first developing a computational model of the cerebellar microcircuit involved in the acquisition of conditioned reflexes and training it with synthetic data generated based on physiological recordings. Secondly, the cerebellar model is interfaced with the brain of an anesthetized rat, connecting the model’s inputs and outputs to afferent and efferent cerebellar structures. As a result, we show that the anesthetized rat, equipped with our neuroprosthetic system, can be classically conditioned to the acquisition of an eye-blink response. However, non-stationarities in the recorded biological signals limit the performance of the cerebellar model. Thus, we introduce an updated cerebellar model and validate it with physiological recordings showing that learning becomes stable and reliable. The resulting system represents an important step toward replacing lost functions of the central nervous system via neuroprosthetics, obtained by integrating a synthetic circuit with the afferent and efferent pathways of a damaged brain region. These results also embody an early example of science-based medicine, where on the one hand the neuroprosthetic system directly validates a theory of cerebellar learning that informed the design of the system, and on the other one it takes a step toward the development of neuro-prostheses that could recover lost learning functions in animals and, in the longer term, humans.

## Introduction

Neural prostheses between the central nervous system and peripheral systems have a relatively recent development history. Some examples are retinal and cochlear implants (Eddington et al., 1978; Wilson et al., 1991; Zrenner, 2002; Cohen, 2007), and brain computer interface systems controlling artificial limbs (Chapin et al., 1999; Schwartz et al., 2006; Moritz et al., 2008; Hochberg et al., 2012). However, the bi-directional coupling of a prosthetic system with the central nervous system has only very recently been addressed (Berger et al., 2011; Bamford et al., 2012; Giovannucci et al., 2010). Here we demonstrate the functional bi-directional coupling of an artificial system and the central nervous system in the context of classical conditioning. Classical conditioning is one of the most essential forms of associative learning (Pavlov and Anrep, 1927). In classical conditioning, an initially neutral Conditioned Stimulus (CS – see Table A1 in the Appendix for the list of abbreviations) precedes an aversive or appetitive Unconditioned Stimulus (US), leading to the acquisition of a Conditioned Response (CR). A widely employed paradigm in classical conditioning is eye-blink reflex conditioning, where an animal is exposed to a CS, e.g., a tone, followed after a certain inter-stimulus interval (ISI) by an aversive US to the eye or periorbital area, e.g., an air-puff (Kehoe and Macrae, 2002). After repeated paired stimulus presentations, the animal closes the eyelids in anticipation of the expected air-puff; this anticipatory action is known as the conditioned response. If a conditioned animal is subsequently exposed to tones not followed by the air-puff US (CS-alone stimulation or extinction training), the previously acquired associative CR disappears and the CS reacquires its initial neutral nature. Remarkably, if we repeat the initial training after extinction, the CRs are more rapidly acquired, a phenomenon known as *savings* (Napier et al., 1992).

**Figure 1:**
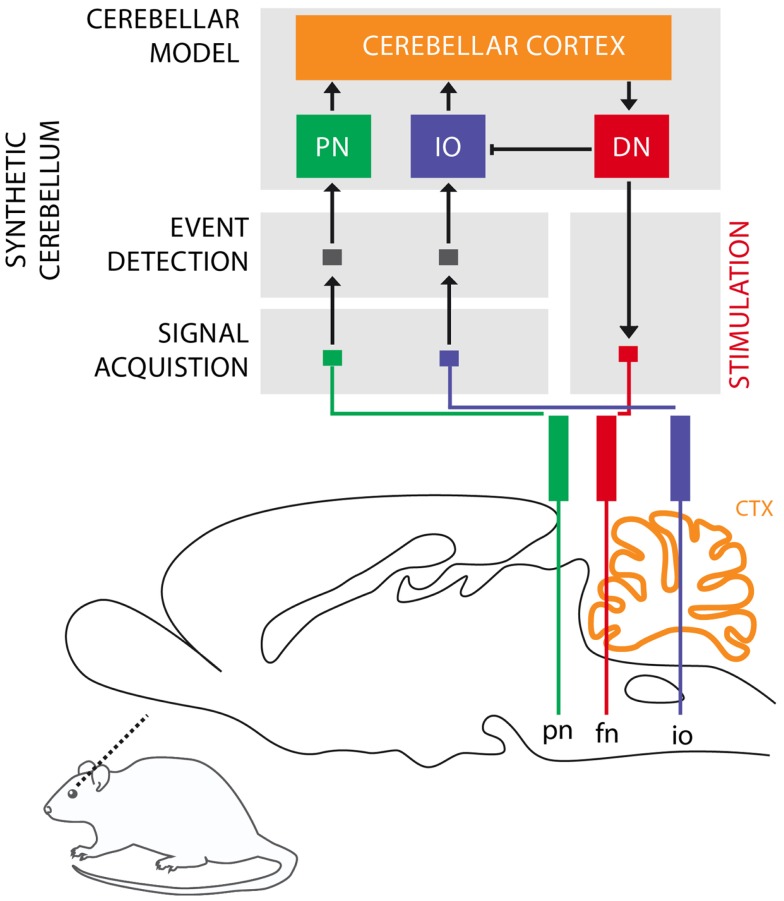
**Biological microcircuit and synthetic counterpart**. Recording (PN and IO) and stimulation sites are shown. After amplification and filtering of the signals recorded in the afferent structures, discrete events retrieved from multi unit activity are isolated by the event detection stages of the system, such that they are fed to their counterparts in the synthetic cerebellum (PN and IO). In the intact circuit, the repeated coincidence of CS and US signals within the cerebellar cortex induces plasticity causing the cerebellum to respond to the CS with a CR. In our model, once such a CR is acquired, it is relayed via the synthetic DN to the facial nucleus (FN) of the rat as an electrical stimulation that causes the animal to trigger the behavioral CR, i.e., the eye-blink. In addition, within the model, the CR triggered by the DN inhibits the IO, preventing a US-derived signal from reaching the cerebellum once a protective action has already been issued. Since anesthesia prevents acquisition in the rodent’s cerebellum, behavioral CRs expressed in the set up studied here are controlled by the synthetic circuit.

The cerebellum is critical for the acquisition of CRs in eye-blink conditioning (Hesslow and Yeo, 2002; Christian and Thompson, 2003). The CS signal reaches the cerebellum through the mossy fibers originating in the Pontine Nuclei (PN), while the US signal is projected through the climbing fibers originating in the Inferior Olive (IO). These two projections converge onto the cerebellar Purkinje cells that control through dis-inhibition of the deep nuclear cells. Deep nuclear neurons synapse with the motor neurons responsible for the production of CRs. Purkinje cells, the sole output of the cerebellar cortex, thus indirectly control the motor neurons with an inverse relationship, they drive CRs by learning to timely reduce their activity in presence of the CS (Jirenhed et al., 2007). These areas of the cerebellar cortex, cerebellar nuclei, and IO regulating the acquisition of conditioned eye-blinks constitute one of the many cerebellar microcircuits, which are considered the elementary and parallel computational units that form the cerebellum (see Figure 1).

Based on these assumptions, we have previously developed a computational model of learning in the cerebellum (Verschure and Mintz, 2001; Hofstotter et al., 2002) that was implemented in a VLSI hardware and tested in a robot learning task (Hofstotter et al., 2004). Here, we show how an updated version of this computational model can be deployed as a prototype of a neuroprosthetic device and interfaced with the brain of a living animal, replacing the function of the animal’s inactivated cerebellum. This experiment is one step in a larger set of experiments to validate the Distributed Adaptive Control theory of mind and brain (DAC) that identifies the cerebellum as a key site for the shaping of self-generated states including discrete actions observed in classical conditioning (Verschure, 2012).

In this case, the computational model is fed not with the artificial signals generated in the robot experiments, but with biosignals acquired through electrodes. Biosignals, unlike the robot input signals, are inherently stochastic both because of recording noise and inherent spiking fluctuations. For instance, the climbing fibers (IO) spiking activity is known to have a 1 Hz baseline activity. Indeed, the features of the experimental recordings presented here match the known IO physiology, showing a baseline firing rate of 0.5–2 Hz (De Zeeuw et al., 1998). Therefore, the encoding of the US in the IO will be very noisy, and will prevent learning if we naïvely use the model in Hofstotter et al. (2004). Hence, our first goal is to update such model in order to cope with biosignals.

Our second goal is to validate the algorithms designed for interfacing and operating a neuroprosthetic system in an *in vivo* bio-hybrid preparation. The system under evaluation (Figure 1) implements a real-time model of cerebellar learning that is driven by signals recorded directly from the PN and IO, detecting CS and US events from these recording channels, respectively. The output of the neuroprosthetic system is linked to a microstimulator targeting the facial nucleus (FN), where proper stimulation would evoke well-timed CRs, with latencies matched to the biological circuit being replaced (see Prueckl et al., 2011a for specifics on the physiology of this experiment). Since in our preparation, the acquisition of natural CRs is precluded by anesthesia, any overt CRs observed in the experiment are the result from associative learning occurring solely within the synthetic system.

Finally, we address a learning stability issue emerged during the *in vivo* testing phase: we detected a non-stationarity in the level of spontaneous activity in the IO channel, and quantified its impact on the model performance. Even though learning can still take place, learning stability is hindered and the possibility of chronic implantation is precluded. We thus implemented a variation of the cerebellar algorithm that is robust under slow non-stationarities, in our case slow fluctuations in the IO activity.

We believe that our approach defines a specific paradigm for the generation of neuroprosthetic systems that evolves following four steps: (1) identify the input and output structures and their encoding, (2) identify the anatomical and physiological principles underlying the computations performed by the target system, (3) integrate steps 1 and 2 with the appropriate signal processing in a single device, and (4) miniaturize the neuroprosthetic system while optimizing its power consumption. Here, we emphasize steps 1–3 since we already previously have demonstrated step 4 (Hofstotter et al., 2004; Bamford et al., 2012).

In summary, with this work we sought to provide further evidence for the fundamental principle underlying our model; namely, that the activity of the IO constitutes a teaching signal that controls the acquisition or extinction of CRs, and that by regulating the IO activity, the nucleo-olivary inhibition (NOI) stabilizes the CRs during paired CS–US training and drives extinction during CS-alone stimulation. Our results provide such evidence, and additionally, they demonstrate at the design level the possibility of realizing long-term, noise-resistant implantable low power neuro-prosthesis supporting the acquisition, retention, and extinction of novel behavior even if the biological substrate has lost its learning capability due to trauma or aging.

## Materials and Methods

### Cerebellar model

#### Latencies

**Figure 2:**
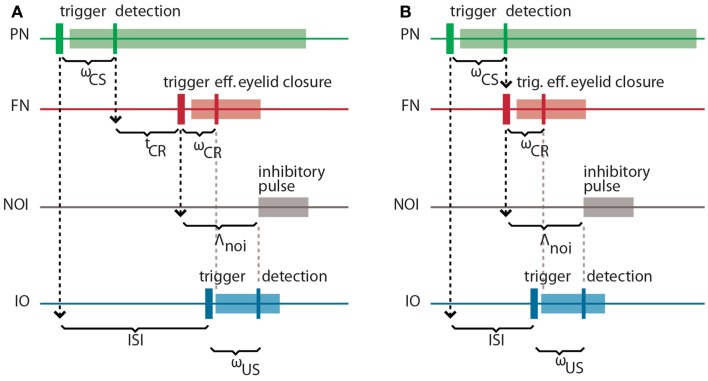
**Intrinsic latencies of the eye-blink conditioning preparation**. **(A)** ISI, inter-stimulus interval; *ω*_CS_, latency between the peripheral CS stimulation and the detection of its associated neuronal response in the PN; *t*_CR_, internal response timing learned by the model between the CS detection and the CR triggering; *ω*_CR_, latency between the neuronal triggering of the CR and the effective eyelid closure, Λ_noi_, delay between the CR trigger and the onset of the negative feedback loop inhibition; *ω*_US_, latency between the US-trigger and the detection of its associated neural response in the IO. **(B)** Same latencies as in **(A)** for the minimum learnable ISI.

It is well-known in the domain of control theory that the latencies and delays inherent in a system to be controlled play an important role in the design of the controller. Here, our controller is based on the cerebellar microcircuit involved in eye-blink conditioning. In nature, such a microcircuit must have internalized the latencies to the eye-blink system in several ways, one of them arguably being through the unusually long latency of the NOI (Hesslow, 1986) that we had previously interpreted as allowing for the matching of the system delays (Hofstotter et al., 2002). Informally, once an error signal reaches the IO, such a delay indicates how far ahead of it the cerebellum should have taken a protective action for it to be effective. Consistently with this view, in the computational model that we employ the latency between the activation of the deep nucleus (DN) and the onset of the inhibition of the IO (the NOI delay, Λ_noi_) sets the anticipation of the CR execution relative to the expected US arrival (Hofstotter et al., 2002) (see Figure 2). Therefore, we will first discuss the latencies associated with the task of classical conditioning, since their specific properties underlie the cerebellar computational model.

Concretely, setting a functional delay for the NOI requires knowledge of the transmission or mechanical latencies involved in the task, otherwise the internal timing of the CR and US signals would result in non-adaptive CRs coming too late or too early with respect to the US air-puff. In other words, for the blink to anticipate the air-puff, Λ_noi_ has to exceed the sum of the sensory latency between the air-puff reaching the cornea and the US detection (*ω*_US_) plus the effector latency between the CR triggering by the DN and the effective eyelid closure (*ω*_CR_):

(1) $$\Lambda_{\text{noi}}\geq \omega_{\text{CR}}+\omega_{\text{US}}.$$

In the literature, this sum of afferent plus efferent latencies is referred to as the delay of the error feedback (Miall et al., 1993). By setting Λ_noi_ to this minimal latency, the CR and the US onsets will match. However, to achieve a better protection form the US, the best temporal arrangement of CR and US is that of the US onset coinciding with the middle of the CR. Given that we elicit the CR by an electrical stimulation lasting 150 ms (Prueckl et al., 2011b), such a temporal arrangement is achieved adding 75 ms to the minimal latency in Eq. (1).

On the other hand, the sum of the latency between the onset of the CS delivery and its detection (*ω*_CS_) plus the latency between the FN stimulation and the CR execution (*ω*_CR_) affects the optimal internal timing (*t*_CR_) that the model has to acquire for a given ISI:

(2) $$t_{\text{CR}}=\text{ISI}-\left(\omega_{\text{CS}}+\omega_{\text{CR}}\right).$$

This time interval is shorter than the external ISI since it accounts for the detection and execution latencies. Note that diminishing *t*_CR_ toward 0, we get the minimum ISI that is learnable by the model, where CS detection immediately triggers a CR:

$${\text{min}}_{\text{ISI}}=\omega_{\text{CS}}+\omega_{\text{CR}}.$$

For an ISI shorter than min_ISI_, a CR initiated by the cerebellum will always come after the US. For this reason, we will design a controller that only acquires CRs whenever the ISI exceeds this value.

#### Computational model

In what follows, we summarize the biological model based on Verschure and Mintz (2001) and Hofstotter et al. (2002) and upgraded to cope with biosignals. Our model is based on the following assumptions:

1. the cerebellum is the brain area principally involved in the acquisition of a CR in the delay classical conditioning paradigm;
2. the only inputs received by the cerebellum are the mossy fibers, carrying CS-related information, and the climbing fibers, carrying US-related information;
3. the mechanism responsible for the acquisition of a conditioned response is plasticity at the parallel fiber to Purkinje cell synapses;
4. such plasticity is induced by the stimulation of parallel fiber, alone (long-term potentiation – LTP) or jointly with climbing fiber (long-term depression – LTD);
5. IO, cerebellar cortex, and DN are organized in distinct micro-complexes, which constitute negative feedback loops over IO;
6. the timing of the CR is adapted to the length of the ISI by these olivo-cortico-nuclear feedback loops that control the plasticity at parallel fiber-Purkinje cell synapses by gating the climbing fiber error signal;
7. the training procedure leads to a pause in Purkinje cell activity following CS presentation;
8. a CR is triggered by dis-inhibition of the deep nucleus by the cessation of Purkinje cell firing;
9. Purkinje cells operate in two distinct modes: a spontaneous and a CS-driven mode. Informally, the Purkinje cell is always maintained active during spontaneous activity of the input parallel fiber. However, during a CS presentation, Purkinje cells switch to a decaying activity. For a detailed explanation see Hofstotter et al. (2004).

Here, in order to deploy the cerebellar model on a low power a VLSI platform, we generated a computational model functionally equivalent to previous versions (Verschure and Mintz, 2001; Hofstotter et al., 2002, 2004) albeit more abstract from an anatomical standpoint to ensure computational efficiency.

##### Process descriptions

**Figure 3:**
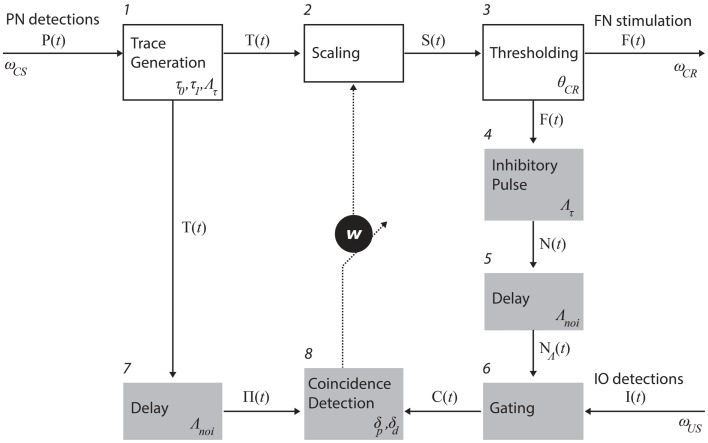
**Functional model of the cerebellum**. The processes in the top row (white boxes) map PN activity into action; in the case of eye-blink conditioning, tone detections into eye-blinks. Such mapping is controlled by the memory parameter *w*. The shaded processes adapt the mapping, namely, they are involved in the adjustment of *w*. The numbers identify specific processes. The latencies affecting each of the recording and stimulating channels as well as the parameters used in each process (see main text for an explanation).

The trace generation, scaling, and thresholding processes (1, 2, and 3 in Figure 3) model the processing of information that enters the cerebellum via the mossy fibers and leaves it through the excitatory axons of deep nuclear cells that projects to red nucleus which, in turn, excites FN (Hesslow and Yeo, 2002; Christian and Thompson, 2003). The trace generation (1) process codes the time since the CS onset with a decaying trace having a fixed initial value (*τ*_0_), final value (*τ*_1_), and duration (Λ*_τ_*). This trace defines the memory span of the system; i.e., the maximum temporal gap between CS detection and a CR execution learnable by the system. The Scaling (2) process multiplies the trace with the memory parameter *w*, which is the only parameter modified by learning. With *w*, we mimic the changes in synaptic efficacy that occur in the molecular layer, due to LTD in the parallel fiber to Purkinje cell synapse and/or other kinds of associative plasticity (Dean et al., 2010). Lastly, the thresholding (3) process triggers a CR whenever the value of the scaled trace falls below a decision threshold (*θ*_CR_). Within this process, we collapse all the transductions that occur postsynaptically from the Purkinje cells down to the efferents of the deep nuclei. In short, these three processes map event detections in the PN into stimulation of the rat FN. The parameter *w* regulates the mapping and, by scaling the trace signal, controls whether a response is triggered or not, and if so, determines its timing in a way analogous to the biological system.

Three processes control the negative feedback loop that stabilizes learning: inhibitory pulse (4), delay (5), and gating (6). The role of the negative feedback in classical conditioning is to prevent the error signal triggered by the US from reaching the cerebellar cortex if a CR has already been triggered (Medina et al., 2001; Verschure and Mintz, 2001). Processes 4 and 5 set the shape of the inhibitory square pulse. Its duration matches the duration of the CS trace, such that the IO can be inhibited for the whole duration of the CS trace. Process 4 delays the pulse by Λ_noi_ seconds. In practice, the value used was on the order of 100 ms. The gating process (6) suppresses IO detections that co-occur with the inhibitory pulse. In summary, these components functionally reproduce the inhibitory control of the deep nuclear cells over the IO (Bengtsson and Hesslow, 2006).

The last two components, delay (7) and coincidence detection (8), update the associative weight *w*, thereby controlling the learning of the CR timing. Process delay delays the CS stimulus trace by Λ_noi_ seconds (same value introduced above). The resulting trace defines the temporal window wherein errors detected by IO can be prevented by the cerebellum. For instance, if a US is detected following a CS but ahead of this temporal window, then the CS–US interval is too short and any CR initiated by the cerebellum after the CS detection could not avoid the US [see Equation (1)]. Likewise, such a trace defines a so-called eligibility window wherein IO activity can be associated with a given PN detection. In short, the system has a minimum ISI of Λ_noi_ seconds, and a maximum ISI of Λ_noi_ + Λ*_τ_* seconds. Lastly, the coincidence detection process (8) checks whether event detections in the CS and US pathways coincide and updates *w* accordingly. Namely, it decreases *w* every time an IO detection overlaps with the eligibility trace, and increases *w* if no IO detection occurs during that period. The function performed by these last two processes mimics the control of plasticity in the parallel fiber-Purkinje cell synapse (Wang et al., 2000; Safo and Regehr, 2008; Sarkisov and Wang, 2008). The initial value of *w* is set to *w*_0_.

#### Calibration of the cerebellar model

##### Definition of the optimization problem

We mentioned that the coincidence detection process in Figure 3 modulates the *w* parameter thereby controlling the acquisition and timing of CRs. In our implementation, the synaptic efficacy *w* is modified in linear steps, namely, *δ_d_* for depression and *δ_p_* for potentiation. The cerebellar model optimization consists of selecting the plasticity parameters *δ_d_* and *δ_p_* that result in a desired learning behavior. We solve this optimization problem in two different scenarios: with synthetic data or with data directly obtained from the brain activity of the animal. With the former, we assess the properties of the model, whereas with the latter it is applied in the bio-hybrid preparation. In both cases, the data consist of a set of detections in both recording sites of the system (PN and IO) and each set might contain evoked-detections (caused by the CS or the US, respectively) of spontaneous events. We refer to the former as true detections (TDs) and to the second as false alarms (FAs).

Informally, we impose that the learning dynamics of the system mirror the behavior: when CSs and USs are paired, the circuit should learn to produce CRs within tens of trials; when in a trained animal CSs are not paired with USs, the circuit should unlearn to produce CRs within tens of trials; all other conditions should not alter the circuit transfer function. More formally, the optimization problem is described by a linear system representing three types of constraints: acquisition, extinction, and stability (see Table 1).

**Table 1:** **Stimulation conditions for. the closed-loop experiment**.

ID	Experimental condition	Description
1	Acquisition	Paired CS–US presentation leading to acquisition of CRs
2	Extinction	CS-alone trials with CR leading to extinction of CRs
3	Stability	CS-alone or unpaired CS–US trials with no CR, causing no modification of the memory parameter

*See text for further explanation*.

##### Estimation of plasticity events

PN (CS) and IO (US) detected or artificially generated events are coded in binary vectors **P** and **I**, where each element is a time step and a value of 1 signals an event. The vector of eligibility traces (**Π**, Box 1 in Figure 3) is obtained by convolving **P** with the eligibility trace waveform (*ϵ*):

Π=P×ϵ,

where *ϵ* is a rectangular pulse lasting Λ*_τ_* seconds and delayed by Λ_noi_ seconds. Here, we fixed these values to 0.3 and 0.1 s, respectively. The first value is in good accordance with the maximum interval between CS and US bridged by the cerebellum in eye-blink conditioning (Moyer et al., 1990; Kalmbach et al., 2009), whereas the second matches the most effective interval between parallel fiber and climbing fiber stimulation for the induction of cerebellar LTD (Wang et al., 2000; Safo and Regehr, 2008; Sarkisov and Wang, 2008).

Plasticity events occur under the conditions specified in Table 2. Firstly, a necessary condition for a potentiation or depression event to occur at a given time step (*t*) is that the eligibility trace is non-zero. Secondly, the number of potentiation events is P=∑Π for potentiation occurs for every time step with a non-zero CS eligibility trace. Thirdly, depression occurs when US detection overlaps with the eligibility trace. Hence, the number of depression events can be obtained with the scalar product of **Π** and **I**:

(3) $$D=\Pi^{T}I.$$

**Table 2:** **Plasticity conditions**.

Eligibility trace vector	US vector	Plasticity
**Π**(*t*) = 1	**I**(*t*) = 1	Depression
**Π**(*t*) = 1	any **I**(*t*)	Potentiation
**Π**(*t*) = 0	any **I**(*t*)	No plasticity event

*See text for further explanation*.

Note that whenever a depression event occurs, it outweighs the default potentiation events triggered by the plasticity trace **Π**, resulting in a net depression.

In the presence of CRs, *D* must be corrected to account for the IO events (spontaneous or US-evoked) suppressed by the NOI. Note that since the timing of inhibition depends on the triggering of the CR and the eligibility window is anchored to the CS, rapidly elicited CRs are more effective in gating plasticity than late CRs. In other words, the effectiveness of the gating decreases as the CRs become more delayed. We can heuristically approximate the reduction in IO events reaching the coincidence detection by multiplying the number of IO detections by an estimated *mean* proportion of IO events *not* suppressed by the NOI ($\bar{\sigma }$),

(4) $$D=\Pi^{T}\left(\bar{\sigma }I\right)=\bar{\sigma }\Pi^{T}I,$$

where $\bar{\sigma }=1-\sigma$, with *σ* being the proportion of IO events suppressed by inhibition.

As the equation illustrates, this result can be computed simply by multiplying the result of Equation. (3) by the factor $\bar{\sigma }$.

##### Optimization of the plasticity parameters

At this point, having estimated the number of plasticity events produced by two sets of event detections in PN and IO, we obtain the optimal plasticity parameters (*δ_p_* and *δ_d_*) by solving with weighted least squares of the following system:

(5) $$\left[\begin{array}{cc} {\bar{P}}_{1} & {\bar{D}}_{1}\\ {\bar{P}}_{2} & {\bar{D}}_{2}\\ {\bar{P}}_{3} & {\bar{D}}_{3} \end{array}\right]\left[\begin{array}{c} \delta_{p}\\ -\delta_{d} \end{array}\right]=\left[\begin{array}{c} -\Delta_{a}∕T_{a}\\ \Delta_{e}∕T_{e}\\ 0 \end{array}\right],$$

${\bar{P}}_{i}$ and ${\bar{D}}_{i}$ are the mean plasticity events per trial, potentiation and depression, respectively, and the sub-indexes indicate the experimental condition (see Table 1). They are obtained by dividing **D** and **P** by the number of trials contained in the training set. Δ*_a_* is an estimate of the change in *w* necessary for acquisition and *T_a_* sets the desired number of trials for acquisition. These two values set a target mean change of *w* per trial. For instance, if the initial value of *w* is 0.5 and we estimate that well-timed CRs occur when *w* reaches a value of 0.3, then we set Δ*_a_* to 0.2. Δ*_e_* and *T_e_* are the same values applied to extinction. As we declared in the assumptions of the biological model, and for consistency with classical cerebellar learning theory, that links learning in the cerebellum with LTD in parallel fiber-Purkinje cell synapses (Albus, 1971; Ito et al., 1982), we suppose that CR acquisition requires depression (decrease) of the value of *w* and extinction, a potentiation (increase). Regarding the optimization algorithm, we weighted more the stability constraint since it by itself guarantees the convergence of the learning dynamics, i.e., paired CS–US stimulation yields acquisition and CS-alone stimulation yields extinction. Informally, if under spontaneous IO activity *w* has an average of 0 drift, then an increase in IO activity will lead to acquisition and a decrease, to extinction. Once this constraint is satisfied, the acquisition and extinction constraints modulate the rate of either learning process.

#### Adaptive calibration of the model

In the previous section, we have introduced a calibration method that assumes stationary biosignals during the experiment. Crucially, this is a strong assumption that will hardly ever be met under *in vivo* conditions. In our case, for instance, the rate of IO activity in the bio-hybrid experiment markedly fluctuated producing non-associative modifications in the synaptic efficacy *w*. For this reason, here we introduce an adaptive version of the calibration method that supports non-stationary responses in IO activity. Since the recalibration has to occur without resorting to additional training data, we keep the same acquisition and extinction constraints used for the initial calibration, and we only update the stability constraint, introducing in this constraint the current estimation of the rate of spontaneous IO activity.

The recalibration is periodically performed, with a fixed time interval. In the experiment, we used 150 s that corresponds roughly to 10 trials. Such recalibration requires an estimate of the ongoing level of spontaneous activity in the IO (IO_far_), where the sub-index far stands for the false alarm rate. To compute this estimate we count the number of IO detections between recalibrations. Note that, since the system is blind to whether the detections are spontaneous or evoked, i.e., it has no knowledge whether stimuli are presented or not, for the estimation of IO_far_, all detections are considered spontaneous. During acquisition, given that some of the IO detections will be US-evoked, this results in an over-estimation of the true IO_far_: the estimate is more accurate for a higher proportion of spontaneous detections to evoked ones, a result that can be easily achieved using large inter-trial intervals (ITI).

Since the IO rate only affects the number of depression events, only ${\bar{D}}_{3}$ in Equation (5) (accounting for the number of depression events during spontaneous activity) has to be updated, whereas the other term has no dependence on IO activity. By updating regularly such parameter, we provide an algorithm simple enough to be implemented in a low power VLSI solution. Indeed, algebraic manipulation shows that we can compute the solution to the optimization for each of the two plasticity parameters as a ratio of two polynomials with a maximum degree of 1 (for the derivation see Appendix). For instance, in the case of δ*_p_* we have:

$$\delta_{p}=\frac{\alpha_{2}{\text{IO}}_{\text{far}}^{2}+\alpha_{1}\text{I}{\text{O}}_{\text{far}}+\alpha_{0}}{\beta_{2}{\text{IO}}_{\text{far}}^{2}+\beta_{1}\text{I}{\text{O}}_{\text{far}}+\beta_{0}},$$

where the coefficients of the polynomials are determined only by the training data. For the detailed derivation of this formula, see Appendix 2.

### Work-flow of the bio-hybrid experiment

The methods introduced so far were common to the simulation and *in vivo* experiments. In what follows we introduce the methodology specifically developed for the bio-hybrid preparation.

#### Animal preparation and recordings

The experimental procedure has been previously described in Prueckl et al. (2011a). In summary, the bio-hybrid experiment was performed on one naïve male Sprague Dawley rat. The rat was housed in a cage with ad lib food and water under a reversed 12 h dark/light cycle. On the experiment day, the rat was anesthetized with i.p. injection of ketamine hydrochloride (100 mg/kg) and xylazine (5 mg/kg) mixture. Body temperature was maintained by a heating pad set to 37°C and supplementary doses of Xylazine and Ketamine were administered as required. The rat’s head was positioned in a stereotaxic head holder (Kopf instruments). The skull was exposed, connective tissue was removed, and skull was dried using a H_2_O_2_ solution (30%). Openings were drilled in the skull over the left IO and PN to allow the insertion of recording electrodes. The electrodes – a 5 Mohm tungsten electrode (A-M Systems, WA, USA) for the IO, and a 10-channel titanium-nitride micro electrode array (Faculty of Engineering, Tel Aviv University) for the PN – were lowered vertically into the brainstem until a reliable response to tone (PN) and air-puff (IO) was observed. Signals from both recording sites were band-pass filtered (300–3000 kHz) to work in the multi unit activity range. A stimulating electrode was also placed in the facial nucleus and tested to induce reliable eye-blinks when 200 mA 0.1 ms constant-current pulses with a frequency of 80 Hz for 150 ms were delivered.

Once the experiment was completed, a direct current was passed through the electrodes (0.5 mA for the IO and 1 mA for the PN), the rat was sacrificed with an overdose of pentobarbital and perfused transcardially (0.9% saline followed by 10% formalin solution), the brain was removed, and sliced into 50 μm coronal sections, stained with thionine blue, and electrode locations were confirmed under a light microscope. All procedures were approved by the Tel Aviv University Animal Care and Use Committee (P-05-004).

#### Experimental protocol

For the classical conditioning preparation, we used as the CS a white-noise stimulus at 67–70 dB with a duration of 450 ms and a 150 ms on air-puff as the US. The presence of CRs was verified by recording the electromyography from the orbicularis oculi. The inter-stimulus interval was set to 300 ms and the ITI was randomized between 10 and 15 s.

After validating the responsiveness of the multi unit activity signal to the air-puff and the tone by visual inspection of both peri-stimulus time histograms of multi unit activity events, we recorded a training data-set that was comprised of 30 trials with paired CS–US presentation, followed by 2 min of spontaneous activity. This data-set was then used to calibrate the synthetic cerebellum (see below). After calibration we proceeded with the classical conditioning paradigm, presenting the animal with paired CS–US stimulations until stable CRs are observed. Subsequently, CS-alone presentations were delivered until extinction of the CRs has been achieved.

#### Signal processing

The goal of the signal processing stage is to detect in the multi unit activity signal the onset of the responses to the CS and US, i.e., to the tone and the air-puff stimuli, respectively. Given the intended implementation in a low power VLSI solution, we limit ourselves to low complexity algorithms. Briefly, we detect sustained increases in the variability of the multi unit activity signal occurring after each stimulus presentation. This is achieved with the following steps: first we subtract from the signal a running estimation of the mean and rectify the resulting signal. Secondly, the signal is smoothed to obtain a short-term temporal average that serves as a measure monotonically related to the variability increase. Lastly, event detection occurs every time the resulting variability signal surpasses a certain detection threshold.

We *a priori* defined the windows of possible true detections (TDs) for each channel (10–150 ms after the trigger in the PN and 5–205 ms after the trigger in the IO). Likewise, the performance of the signal detection can be summarized with the true detections ratio (TDR) and the false alarm rate (FAR), where the TDR indicates the proportion of stimuli raising *at least* one detection within the TD window, and the FAR the frequency of events detections during the periods of no stimulation, i.e., outside the TD window. Note that since in the IO we found an FAR between 0.5 and 2 Hz, we can compare the IO_far_ with the spontaneous levels of activity in the IO (Jirenhed et al., 2007).

#### Estimation of the event detections

With the calibration data-set, we estimate for both channels the detection performance during early acquisition trials (before any CR is triggered). To estimate the number of detections during CS-alone trials, we combine the PN data from the paired stimulation trials with IO data from the spontaneous activity period. This is done in order not to excessively extend the calibration phase.

#### Optimization of the signal detection regimes

To signal an event detection, we have to first set a detection threshold. Such selection poses a multi-objective optimization problem since we want to simultaneously maximize the TDs and minimize the false alarms, and we do not know *a priori*, which is the best trade-off of both measures that maximizes the chance of success in our experiment. To overcome this problem, we iterate the optimization process over a set of threshold configurations for both input channels, and then select *a posteriori* the one yielding the minimum error in the optimization of the plasticity parameters [Equation (5)]. Likewise, the optimization process, and not us, selects the optimum trade-off between TDs and false alarms. Note that the simplicity of the calibration method previously introduced allows us to iterate over a great number of possible threshold configurations in very little time.

### Use of the model with simulated data

Before deploying the computational model in the bio-hybrid setup, we tested the performance of the model with artificially generated data. To this end, we generate a set of detections for each channel according to a certain pair of TDR and FAR statistics. From these two statistics, we produce the binary vectors of detections **P** and **I**, and for this we convert the TDR and FAR to event probabilities per time step. We obtain the probability of detection in the absence of stimulation by multiplying the FAR by the model time step, which is 2 ms. Regarding TDs, to convert the TDR to a probability we have to consider the size of the TD window. Operationally, since we interpret the TDR as the probability of getting *at least* one event within the detection window, we have to find the event detection probability yielding no events during the TD window with a probability of 1 − TDR. This event detection probability is equal to $1-\sqrt[{n}]{1-\text{TDR}}$, where *n* is the size of the TD window in time steps.

## Results

### Simulation experiments

#### Performance of the model with spontaneous activity in the IO

**Figure 4:**
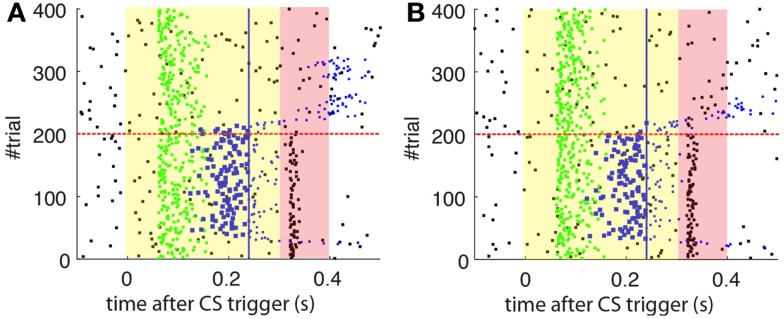
**Raster plots of the inputs and outputs of the model with and without stability constraint**. **(A)** Model with stability constraint. PN detections (green), IO detections (black), and CR triggers (blue, well-timed thick, and late thin). CS (yellow area) and US (pink area) periods. The horizontal dashed red line separates acquisition and extinction phases. Vertical blue line marks the limit for well-timed CRs. **(B)** Model without stability constraint. Data plotted as in**(A)**.

**Figure 5:**
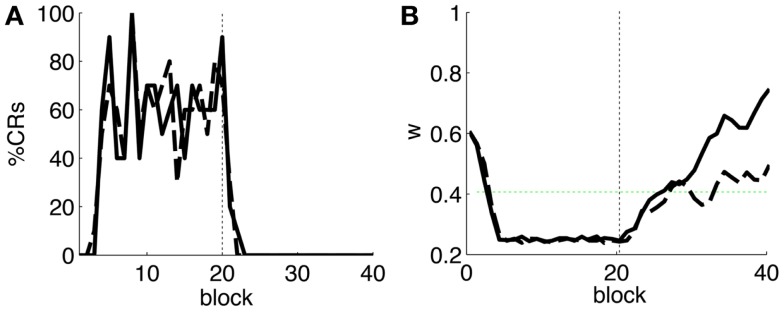
**Behavior of the model with simulated data**. **(A)** Behavioral performance. Percentage of CRs per block of trials of the model fitted with stability constraint (solid line) and without (dashed line). The vertical dotted line separates acquisition and extinction training. **(B)** Trajectory of *w* in the model fitted with stability constraint (dashed line) and without (solid line). The horizontal green dotted line marks the level above which the model does not trigger any CRs. Blocks of 10 trials.

As a first step, we test whether our model supports the acquisition and extinction of CRs when the IO displays spontaneous activity (see parameters in Table 3). The outcome of a representative simulation shows that indeed the model adapts well to the case of baseline IO activity (Figure 4A). It acquires well-timed CRs in CS–US paired trials and extinguishes them in CS-alone unpaired trials (Figures 4A and 5A), and, importantly, the parameter *w* reaches a stable plateau after complete extinction (Figure 5B). We stress that the stabilization occurring at the end of extinction even in the presence of CS-alone stimulations, stems from the stability condition in Equation (5). If we remove this constraint, the overt behavioral results are similar (Figures 4B and 5A) but the underlying memory dynamics differ (Figure 5B). Indeed, behaviorally both models only differ in the extinction phase, which is slower for the model with stability. However, in regard to the model’s state, without stability, the synaptic efficacy *w* grows also after extinction of the CRs has been accomplished. Note that, in consequence this can make reacquisition harder than acquisition if the extinction training is maintained, which goes against the experimental evidence (Kehoe and Macrae, 2002). In conclusion, the computational model of the cerebellum is also functional when the IO has baseline activity, requiring only a proper calibration of the plasticity parameters.

**Table 3:** **Parameters of the simulation**.

Parameter	Value
**CEREBELLAR MODEL**	
CS trace	
*τ*_0_	1
*τ*_1_	0.5
Λ*_τ_*	350 ms
Λ_noi_	100 ms
*θ*_CR_	0.2
*w*_0_	0.5
**SIGNAL DETECTION**	
PN*_td_*	0.95
PN_far_	0 Hz
IO*_td_*	0.75
IO_far_	1.0 Hz
**FITTING CONSTRAINTS**	
Δ*_a_*, Δ*_e_*	0.2
*T_a_*, *T_e_*	40

#### Effect of the latencies of the cerebellar model

We previously discussed the relevance of the latencies in the design of the controller. Here, we will illustrate with two examples the functional implications of the two latencies implemented in the model, namely, the latency of the NOI and the latency of the plasticity trace. We recall that in our model, both latencies are set to the same value, namely, Λ_noi_ seconds.

**Figure 6:**
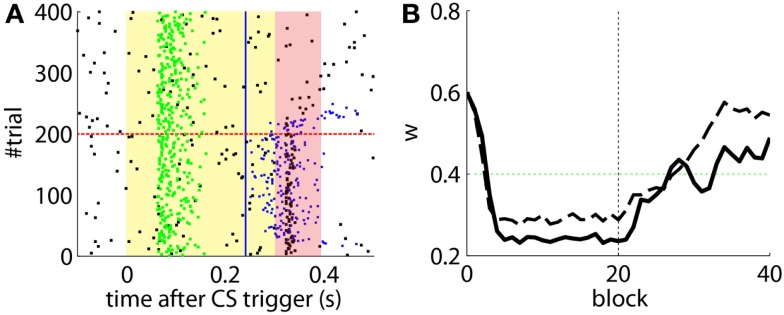
**Results with and without delayed NOI**. **(A)** Raster plot with the output of the model with the delay of the NOI set to 0 s. **(B)** Trajectory of *w* in the model with a delay of 100 ms in the NOI (solid line) and with no delay (dashed line). The horizontal green dotted line marks the level above which the model does not trigger any CRs. Blocks of 10 trials.

**Figure 7:**
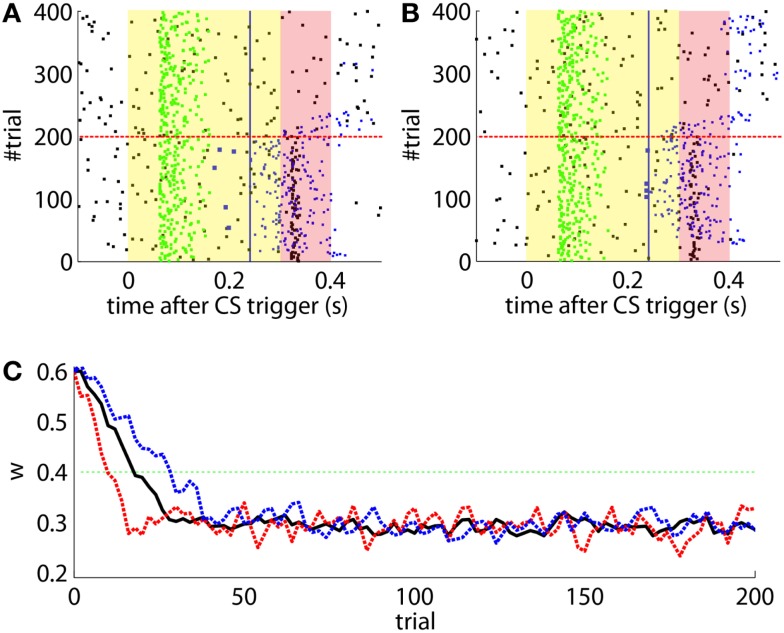
**Results with non-delayed NOI inhibition in different conditions**. Raster plots with the output of the model with the delay of the NOI set to 0 s. **(B)** the model constraint to acquire CRs twice as fast or with **(A)**. A ratio of TDs in the IO lowered to 50%. **(C)** Traces of the synaptic efficacy *w* for the simulation in Figure 6 (black) compared to the simulations in **(A)** (dotted red) and **(B)** (dotted blue).

The effects of the NOI latency on the timing of the CRs have already been discussed in the literature at the theoretical level (Hesslow and Ivarsson, 1994; Hofstotter et al., 2002), and demonstrated in experimental set-ups (Herreros Alonso and Verschure, 2013). Here, and because of the noisy input conditions, we see that if we do not apply any delay to the NOI, the triggered CR eventually anticipates the US, but by too short a latency too be considered effective (Figure 6A). Therefore, even though the model triggers CRs, they are maladaptively timed. Indeed, the synaptic efficacy *w* fails to reach a level sufficiently low to initiate well-timed CRs, as it does when the latency of the NOI is properly set (Figure 6B). Note, however, that the jitter of the trace of the synaptic efficacy *w* occasionally brings the CR triggers close to the criterion of correct timing. Given that, if such a jitter will be increased it would be possible for occasional CRs to anticipate the US sufficiently to be characterized as *well-timed*. This occurs if, for instance, the signal to noise ratio of the IO signal decreases (Figure 7B, with TDs in the IO lowered from 70 to 50%) or if we force the learning to be faster (Figure 7A). This by no means indicates that the model works better if the signal conditions are worse, it only indicates that as the dynamics of the model become more noisy (Figure 7C), some well-timed CRs may incidentally be triggered, even if the delay of the NOI is not correctly set.

**Figure 8:**
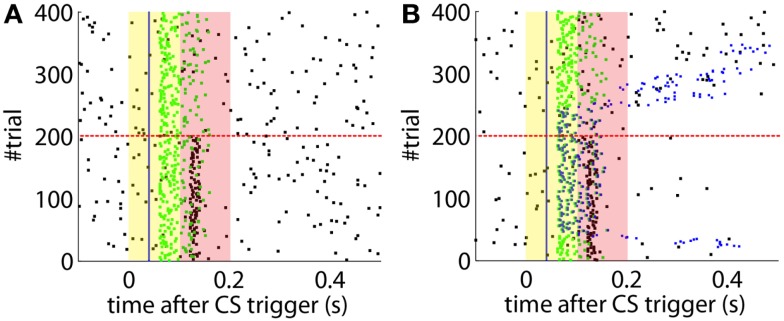
**Effect of the delayed plasticity trace on the behavior**. **(A)** Model with plasticity trace starting *Λ*_noi_ seconds after each PN detection. **(B)** Model with plasticity trace starting right after each PN detection. Data plotted as inFigure 4.

Not delaying the plasticity trace leads to a different kind of non-adaptive behavior. In this case, if we set an ISI below the minimum ISI described in Equation (1), the computational model without delayed plasticity acquires CRs that can only be late CRs by definition (Figure 8B). In contrast, setting the appropriate delay to the plasticity trace avoids building any association between CS and US that are too close in time (Figure 8A).

### Bio-hybrid experiment

#### Evaluation of the training data

**Figure 9:**
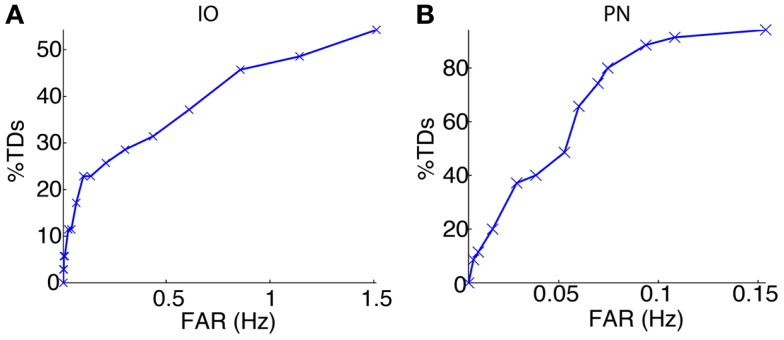
**Event detection performance for the recording sites**. ROC curves for the IO **(A)** and the PN **(B)** event detections.

We started the bio-hybrid experiment recording a training data-set composed of 30 trials of paired CS–US stimulation, with an ISI of 300 ms and an ITI of 10 s. After applying the signal processing algorithms (see Materials and Methods), we built the receiver operating characteristic (ROC) curve for each of the channels (Figure 9). The PN channel TDs reached 100% with a false alarm rate close to 0.1 Hz while the IO displayed TDs near 50% for the range of optimal FARs (~1 Hz). Therefore the PN channel was reliable while the IO channel was poor from the detection standpoint.

#### Optimization of the plasticity parameters and signal detection regimes

The following phase entailed tuning the plasticity parameters and selecting the optimal signal detection thresholds. The optimization process selected detection thresholds yielding a percentage of TDs of 48.6% and a FAR of 1.14 Hz for the IO channel, and a 91.4% of TDs with a FAR of 0.11 Hz for the PN.

The model calibration sets the potentiation and depression steps (*δ_p_* and *δ_d_*) to 0.0161 and 3.36e−5, respectively.

**Figure 10:**
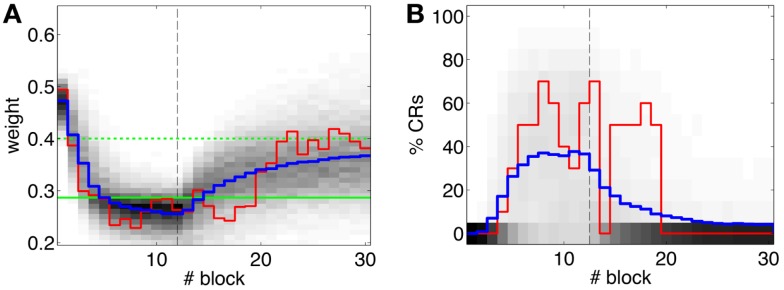
**Performance of the experiment predicted by the training data**. **(A)** Trajectory of the memory parameter after 2500 simulations plotted in blocks of 10 trials. The simulated experiment contained 120 trials of acquisition and 180 trials of extinction. Distribution of the block-by-block values of *w* (grayscale) with mean (blue) and output of a sample simulation (red) are shown. We indicate the levels of the weight that result in late (upper green line) and well-timed CRs (lower green line). The transition from acquisition to extinction training is marked by a vertical line. **(B)** Predicted behavioral performance after 2500 simulations. Percentage of well-timed CRs. Distribution of the block-by-block performance (grayscale) with mean (blue) and result (red) of a sample simulation [same as in**(A)**].

The offline simulation parameterized with the previous values is shown in Figure 10. Firstly, on average acquisition occurs in 40 trials with an asymptotic performance of 40% well-timed CRs. Secondly, there is low chance of obtaining total extinction after 120 trials of CS-alone stimulation. Thus, the simulations predict that a low detection quality in the IO channel may hinder extinction.

#### Evaluation of the bio-hybrid experiment

**Figure 11:**
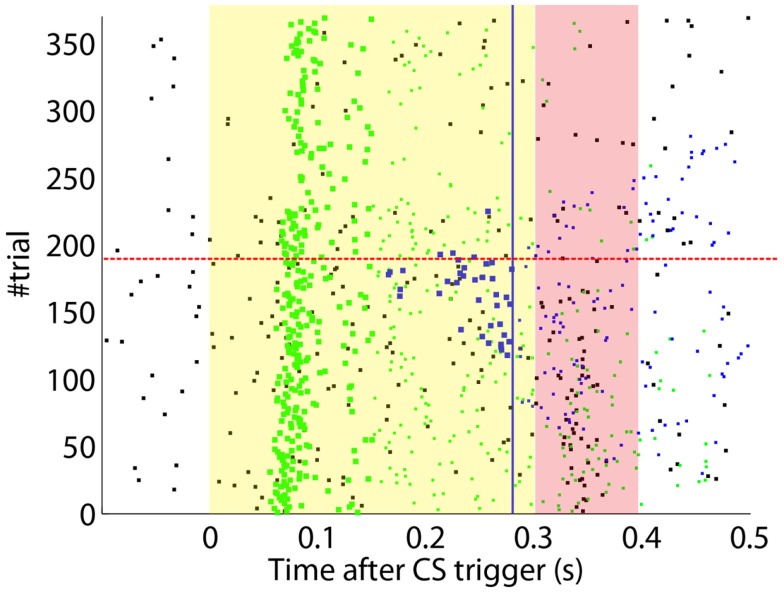
**Event detections and triggers during the online experiment**. Raster plot with the PN detections (blue dots; well-timed PNs are thicker), IO detections (black), and CR triggers (blue dots; well-timed triggers are thicker). The CS (yellow area) and US (pink area) periods are indicated. Blue line separates well-timed from late CRs. The horizontal dashed red line separates acquisition and extinction phases.

After the preliminary assessment of the quality of the biosignals, we proceeded with the online classical conditioning experiment (Figure 11). The experiment lasted 1 h 20 min and comprised 190 CS–US stimulation trials (acquisition) followed by 180 CS-alone trials (extinction), with randomized ITIs between 10 and 15 s. In Figure 11, we display events detected and stimulations triggered by the neuroprosthetic system. For the whole experiment, in the PN there was a TDR in of 75.5% and a FAR of 0.1 Hz that include a high number of late CS-detections (Figure 11). Notably, the number of PN detections during baseline was very low (only five false alarms in 80 min). In the IO, we obtained a TDR of 38% and a FAR of 1.2 Hz.

**Figure 12:**
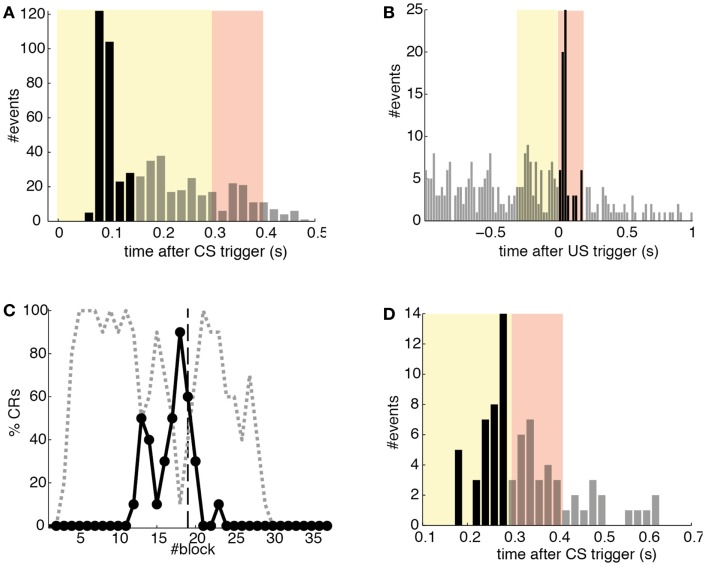
**Quantitative results**. **(A)** Events detected in the PN. Histogram of PN detections relative to the CS-trigger: TDs (black bars) and FAs (gray bars); in this case all FAs are late CS-detections. CS period (yellow area) and US period (pink area). **(B)** Events detected in the IO. Detections in the IO sorted relative to the US-trigger. Data plotted as in **(A)**. **(C)** Behavioral performance of the bio-hybrid. Percentage of well-timed CRs during acquisition and extinction (solid line) are shown. CRs that were not triggered at least 20 ms ahead of the US-trigger appear as late CRs (dashed line). Each block contains 10 trials. **(D)** Timing of CRs. Histogram of the CRs: well-timed (black bars) and late ones (gray bars). CS period (yellow area) and US period (pink area) are indicated. The information is extracted from trials 118 to 190.

Detections in both channels were delayed by tens of milliseconds with respect to the stimulus trigger. The mean latency of the TDs in the PN (*ω*_CS_) was of 96.2 ms after the CS-trigger (Figure 12A) whereas the mean latency in the IO channel (*ω*_US_) was of 68.5 ms (Figure 12B).

The experiment was successful in terms of behavior: well-timed CRs were triggered with regularity toward the end of the acquisition phase, and no CR was triggered during the last 90 trials of the extinction training (Figures 11 and 12C). The first response appeared at trial 29, but the first well-timed CR came only at trial 118. Notice that toward the end of acquisition, the series of well-timed CRs appeared regular. After the onset of the extinction trials (trial 191), well-timed CRs were rapidly extinguished. A block-by-block analysis reveals that the performance fluctuated during acquisition (Figure 12C) and that extinction of well-timed CRs was very rapid, in total there are only four well-timed CRs during extinction, the last one appearing at trial 220, i.e., after 30 trials of extinction. However, the extinction of late CRs was more gradual, encompassing blocks 21–29, i.e., total extinction required 100 trials. No CR was triggered by the system in the last 60 trials of the experiment. Regarding the timing, well-timed CRs occurred on average 50 ms ahead of the US-trigger (Figure 12D).

**Figure 13:**
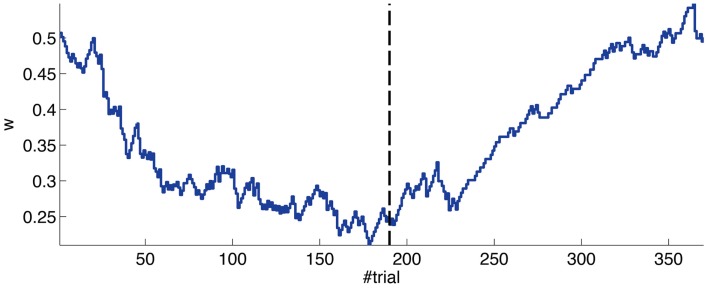
**Weight trajectory during the experiment**. The dashed vertical line separates the acquisition and extinction phases.

The evolution of the synaptic efficacy *w* is displayed in Figure 13. We estimate that given our setup CRs follow a PN event whenever the value of *w* goes below 0.4. However, for such CRs to be anticipatory, *w* should settle at 0.28 or below. During the experiment, *w* decreased steadily during the first 60 trials, down to a value of 0.29. Afterward, the decrease decelerated. The dynamics of *w* suggest that learning has reached an asymptotic-level by the end of the acquisition stage (Figure 13). The mean value of *w* during well-timed CRs was 0.25, corresponding to CRs triggered on average 140 ms after the PN detection. Thus, for an ISI of 300 ms the model acquired an internal timing (*t*_CR_) of 140 ms.

Thus, in conclusion, at a first level of analysis, the results of the bio-hybrid experiment were correct both from the behavioral point of view and also regarding the dynamics of the underlying memory parameter stored in the synthetic cerebellum.

#### Instability of the activity during the recording

Having said that, there were two major caveats in the experiment. First, due to an artifact introduced by the electrical stimulation of the CR, the signal of the IO was masked for 2 s after each CR. This masking was performed at the signal acquisition stage (Prueckl et al., 2011b). For this reason, no events reached the computational model of the cerebellum for 2 s after each CR trigger. Under such circumstances, the model’s NOI became superfluous, because for all its extent there was no IO detection to be inhibited. Or, in other words, the mask at the signal acquisition stage acted as a NOI with 0 latency and longer duration. We have already argued that the latency of the NOI is necessary for consistently achieving a correct timing of the CRs (Figure 6). Thus, in summary, on the one hand, it is reasonable to assume that the well-timed CRs were in part a consequence of the noisy conditions of the input setup (e.g., a IO*_td_* of 38%), and on the other, it is also reasonable to expect that in the absence of the stimulation artifact, the synthetic cerebellum would have achieved a higher proportion of well-timed CRs.

**Figure 14:**
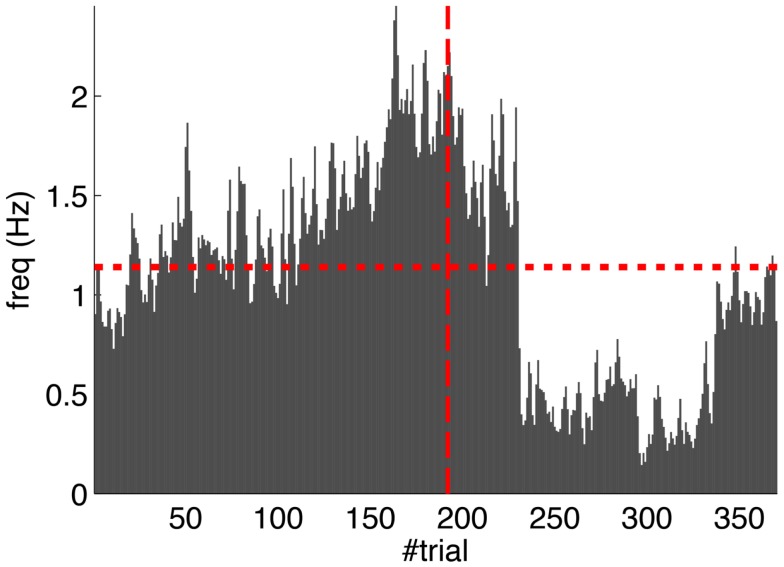
**Fluctuations in the spontaneous IO rate**. Mean IO rate in each trial of the experiment. The horizontal dotted line marks the 1.14 Hz level of activity. The vertical dashed line marks the transition from acquisition to extinction trials.

Second, our calibration of the cerebellar model assumes that the level of spontaneous activity in the IO remains constant. If the IO spontaneous activity significantly deviates from the one estimated during calibration, then *w* will drift, eventually leading to either acquisition or extinction. Since during the conditioning experiment we observed that the spontaneous activity fluctuated (Figure 14), there is the possibility that the behavior observed did not result from associative learning but from changes in *w* due to oscillations in the IO spontaneous activity. In particular, given that the IO spontaneous activity increased during acquisition and decreased during extinction, such fluctuations might have caused or favored the behavioral result.

**Figure 15:**
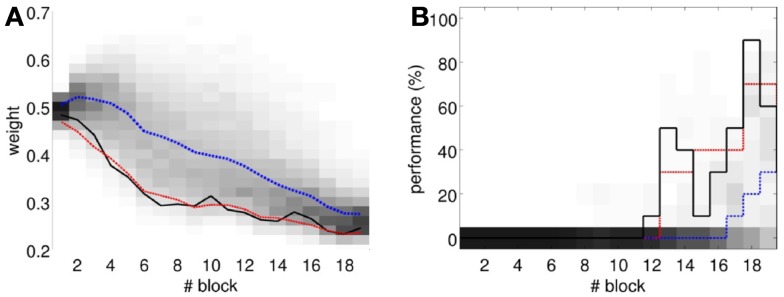
**Observed performance vs. performance during simulated unpaired acquisition**. **(A)** Acquisition during paired CS–US training versus simulated unpaired CS–US. Trajectory of the weight during the acquisition phase of the experiment (black line) plotted against results of 20,000 simulations of unpaired training. Distribution of the simulation results (grayscale), median (blue dotted line), and the 0.05 bottom of the distribution (red line) is shown. Blocks of 10 trials. **(B)** Behavioral performance during acquisition against performance in the simulations. Percentage of CRs during acquisition in the experiment (black line) plotted against the percentage obtained in the simulations. Distribution of the simulation performances (grayscale), median (blue dotted), and the upper 0.1 of the distribution (red line).

To perform an *a posteriori* control for this, we checked whether the observed oscillations in spontaneous activity may lead to acquisition by themselves even in the presence of temporally unrelated CSs and USs. We tested this by simulating unpaired CS–US presentations. For this, we generate experiments with shuffled IO detections within each trial. After performing 20,000 simulations, we observed that the increased spontaneous activity of the IO causes a decrement in *w* during the acquisition phase for *unpaired* stimulation (Figure 15A). Considering the behavior (Figure 15B) in the average simulation, the decrease of *w* yielded to the triggering of a small number of CRs. These results both at the level of the memory parameter and the behavior were clearly below the performance observed on the bio-hybrid experiment but demonstrated nonetheless that under experimental conditions with big variability in the recorded signals, the model might acquire spurious associations.

### Calibration method adapted to fluctuating IO activity

Lastly, we tackle the problem of the instability of the IO activity evidenced in the previous section. For this we apply the adaptive calibration method (see Materials and Methods). We test this method with data from the bio-hybrid preparation, aiming to show that with the adaptive calibration, the cerebellar model becomes robust against slow fluctuations in the baseline IO activity. As a caveat, notice however, that in the bio-hybrid experiment, by definition of a *closed-loop* setup, the data recorded during the session depended on the output of the model. In this case, the data recorded were affected by the electrical stimulation in the FN driving the CR. Thus, to cancel out this effect we replaced the 3 s of the IO signal occurring after each CR, by the signal extracted from random trials with no CR.

**Figure 16:**
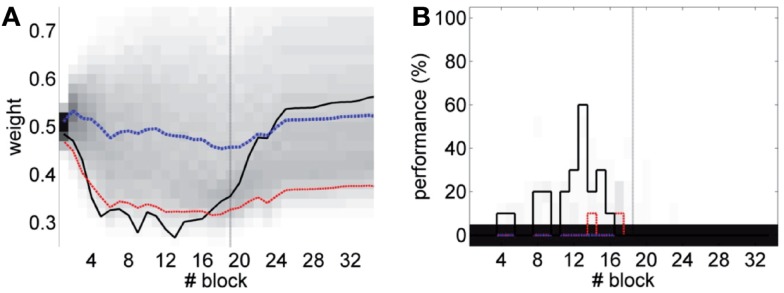
**Performance with the adaptive calibration method**. **(A)** Trajectory of the memory parameter *w* plotted in blocks of 10 trials. Distribution of the performance of the simulations of unpaired CS–US stimulation (grayscale), with mean (dotted blue) and lower 10% (dotted red), for a total of 2500 simulations of 36 blocks. Trajectory of the simulated classical conditioning experiment (solid black), with 18 blocks of acquisition and 18 blocks of extinction. The transition from acquisition to extinction training is marked by a vertical line. **(B)** Behavioral performance of the same simulations. Percentage of well-timed CRs per block is shown. Results plotted with the same convention as in **(A)**.

The results (Figure 16) now clearly separate the performance between the unpaired- and paired-stimulation experiments. Most importantly, there is no acquisition of CRs during unpaired stimulation. In this case, the fluctuations of the baseline IO rate do not push *w* further than ±0.1 from the starting value, both during acquisition and extinction. Regarding the performance, in average there are no well-timed CRs with unpaired CS–US training. On the contrary, in the simulated acquisition and extinction experiment, the overall behavior of acquisition followed by extinction is preserved. In this experiment, the CR performance decreases relative to the result with the bio-hybrid, especially by the end of acquisition, when the recorded IO baseline rate was higher. The reason is that now a high rate of spontaneous detections in the IO diminishes the relative saliency of the US-evoked events, because such a high rate harms rather than helps acquisition. Thus, this simulation confirms that if the same conditions of signal instability of the bio-hybrid experiment are repeated, with the adaptive calibration method we can ensure that any CRs observed will specifically be due to the CS–US association.

## Discussion

In this paper, we have addressed the challenge of defining, interfacing, and validating a neuroprosthetic system for the cerebellum. More in detail, we have: defined a biologically grounded computational model of the circuit targeted for substitution; defined its input and output structures and decoded input events; implemented a prosthetic cerebellum; interfaced it to a rat brain. Our results show that our bio-hybrid preparation shows behaviorally and physiologically valid forms of acquisition and extinction of the conditioned eye-blink response. Our neuroprosthetic system learned to associate a tone with an air-puff, and as result to trigger an anticipatory closure of the eyelid. Since motor CRs are not acquired or expressed under such anesthesia regime, the observed eye-blinks are produced by the synthetic system. The fact that acquired CRs can be abolished by extinction training also indicates that the CRs result from a learning process induced by the contingent association of the CS and the US, thus reproducing the hallmark result of Pavlovian classical conditioning.

Here, we have presented a step towards the enhancement and/or recovery of the capabilities of central nervous system through neuroprosthetic solutions. Recently, another closed-loop solution targeting a different structure of the brain, the hippocampus (Berger et al., 2011) has been presented. This system, however, follows a different approach where firstly the subject had to acquire a specific stimulus response association that was recorded by the neuroprosthetic system, and subsequently the recorded state was used to recover this association after lesion to the hippocampus. Compared to such model, instead of aiming at restoring an acquired memory, our neuroprosthetic aims to fully replace its target structure and to realize the capability to form new memories.

In a parallel effort, the computational model of the cerebellum here presented, together with the signal detection algorithms and the signal acquisition components, have been implemented in a low power VLSI (Bamford et al., 2012). Hence, with the results presented here we provide the feasibility requirements of a neuroprosthetic system, encompassing issues related to stability and non-stationarities.

An earlier version of the computational model presented here was implemented in an aVLSI platform and interfaced with a robot that was conditioned to a visual stimulus predicting a collision (Verschure and Mintz, 2001; Hofstotter et al., 2002, 2004). Thus, after showing that our approach allowed miniaturization and autonomous performance, we have now demonstrated that the model can be applied to the processing of inputs coming from a living brain that are specific to the computation performed, i.e., CS, US, and trigger its specific output: the CR.

In our experiment, the IO channel provided the only teaching signal to the system. This channel displayed a spontaneous level of activity in the 0.5–2 Hz range, i.e., the level of activity expected in a single IO cell. However, in healthy animals, acquisition of an eye-blink CR is controlled by a cerebellar micro-complex, encompassing not one, but a number of IO cells. This imposes different constraints on the learning system because the IO-derived error signal for our neuroprosthetic is in all likelihood much impoverished as compared to its biological counterpart. Hence, we expect that the key feature to strengthen in our approach is the quality and precision of the data acquisition of the biological preparation. For this, we are planning further experiments in a chronic implant together with higher bandwidth physiology.

We reported two major caveats in the experimental preparation: the instability of the IO spontaneous rate, and presence of a stimulation artifact that precluded reliable read-out of the IO signal. Regarding the first problem, in the bio-hybrid experiment we computed the plasticity parameters assuming that the spontaneous IO rate inferred from the calibration data remained stable throughout the experiment. However, we observed that fluctuations in the spontaneous IO firing rate induced a drift in the *w* synaptic weight. Comparing this performance with simulated unpaired CS–US experiments, we saw that the performance with unpaired stimulation tended to be below the one observed in our experiment, but we also saw that the system triggered non-associative CRs. Next, we showed in simulations that with an adaptive calibration method it is possible to compensate for the fluctuations in the IO activity and thus to prevent the acquisition of non-associative CRs (see Figure 16). We can conclude from the bio-hybrid experiment that our silicon cerebellum neuroprosthetic can be tuned to deal with marked fluctuations of its input brain signals. This also demonstrates the robustness of the learning principles implemented in the cerebellum and in particular the negative feedback loop implemented by the DN-IO system.

Additionally, the fluctuations of the IO signal reduced the accuracy of the performance predicted during the calibration step. This justifies the future development of calibration methods accounting for spontaneous drifts in the recorded neural activity.

Regarding the problem of the stimulation artifact, addressing it falls outside the scope of the current analysis. We emphasize that this problem is an issue of engineering of the stimulation system that the biological system does not encounter. We are investigating two solutions. First, given the very short duration of the stimulation pulses (Prueckl et al., 2011b), it is possible to apply a more precise masking to the IO signal, timed to these pulses, that would minimize the signal loss. A second possibility is to avoid electrical stimulation altogether using optogenetics. These aspects need to be taken into account in a next iteration of the neural prosthesis development.

In conclusion, from a bio-engineering perspective we demonstrate that our approach supports outsourcing the acquisition and extinction of an adaptive reflex in an acute preparation to a linked neuroprosthetic system. Given the modularity of the cerebellum, and the common assumption that the cerebellar algorithm performs similar computations throughout its different microcircuits (Marr, 1969; Albus, 1971; Dean et al., 2010), this work could be applied to support other adaptive reflexes as well, as long as their afferent and efferent circuitry could be identified. However, the multifunctionality of a microzone and/or its interactions with other cerebellar microcircuits are not addressed with our approach and would require interconnecting and synchronizing multiple prosthetic microcircuits working in parallel. Additional work is required to reproduce this result with the aVLSI synthetic system, testing this approach with a chronic implant, where one could assess the stability of the acquired memory across days and study long-term bio-compatibility issues.

## Author Contributions

Paul F. M. J. Verschure conceived the experiments. Aryeh H. Taub, Andrea Giovannucci, Ivan Herreros, Roni Hogri, and Robert Prueckl designed and implemented the protocol and setup of the bio-hybrid experiment. Andrea Giovannucci, Ivan Herreros, and Paul F. M. J. Verschure designed the computational model and Andrea Giovannucci, Ivan Herreros, and Robert Prueckl implemented it. The signal processing methods were designed and implemented by Andrea Giovannucci, Sim Bamford, and Robert Prueckl. The linear calibration method was designed and implemented by Ivan Herreros. The adaptive calibration method was designed by Andrea Giovannucci and Ivan Herreros and implemented by Ivan Herreros. Ivan Herreros analyzed the data and ran the simulations. Aryeh H. Taub, Ari Magal, and Roni Hogri performed the physiological experiments from which the neuronal data were acquired. Andrea Giovannucci, Ivan Herreros, and Paul F. M. J. Verschure wrote the paper. Paul F. M. J. Verschure designed the research.

## Conflict of Interest Statement

The authors declare that the research was conducted in the absence of any commercial or financial relationships that could be construed as a potential conflict of interest.

## Appendix

**Table A1:** **List of abbreviations**.

Abbreviation	Meaning
IO	Inferior olive
PN	Pontine nuclei
DN	Deep nuclei
FN	Facial nucleus
NOI	Nucleo-olivary inhibition
CS	Conditioning stimulus
CR	Conditioned response
US	Unconditioned stimulus
UR	Unconditioned response
ISI	Inter-stimulus interval
ITI	Inter-trial interval
Λ_noi_	Latency of the NOI
*δ_p_*	Potentiation step size
*δ_d_*	Depression step size
TDR	Proportion of true detections
FAR	False alarm rate
PN*_td_*	Proportion of true detections in the PN
PN_far_	Rate of false alarms in the PN
IO*_td_*	Proportion of true detections in the IO
IO_far_	Rate of false alarms in the IO
*w*	Synaptic efficacy
*ω*_CS_	Latency between CS-trigger and CS event detection
*ω*_US_	Latency between US-trigger and US-event detection
*ω*_CR_	Latency between CR-trigger and its physical execution
*P_i_*	Number of potentiation events in the condition *i*
${\bar{P}}_{i}$	Mean number of potentiation events per trial in the condition *i*
*D_i_*	Number of depression events in the condition *i*
${\bar{D}}_{i}$	Mean number of depression events per trial in the condition *i*
Δ*_a_*	Estimated amount of change in *w* required for acquisition
Δ*_e_*	Estimated amount of change in *w* required for extinction
*T_a_*	Desired number of trials required for acquisition
*T_e_*	Desired number of trials required for extinction

### 1 Mathematical definition of the cerebellar model

In what follows we provide a compact definition of the cerebellar computational model, describing each of the processes separately. The only additional notation introduced here for convenience are the sample time *s*, that it is used to access the state of a variable in the previous step of the computation, and the Heaviside function, *H*(*x*), defined as:

$$H\left(x\right)=\left\{\begin{array}{cc} 1 & \text{if }x>1\\ 0 & \text{elsewhere} \end{array}\right..$$

The signals and the processes correspond to the ones presented in Figure 3. Note that except **T**(*t*), **S**(*t*), and *w*(*t*), that are real-valued, the rest of the signals are binary.

Process 1: Trace generation.

$$T\left(t\right)=\tau_{0}^{P\left(t\right)}{\left\{H\left(T\left(t-s\right)-\tau_{1}\right)\left(T\left(t-s\right)-\frac{\tau_{0}-\tau_{1}}{\Lambda_{\tau }}\right)\right\}}^{\left(1-P\left(t\right)\right)}.$$

Process 2: Scaling.

S(t)=w(t)T(t).

Process 3: Thresholding.

$$F\left(t\right)=H\left(\theta_{\text{CR}}-S\left(t\right)\right)\;H\left(S\left(t-s\right)-\theta_{\text{CR}}\right).$$

Process 4: Inhibitory pulse.

$$N\left(t\right)=N\left(t-s\right)+F\left(t\right)-F\left(t-\Lambda_{\tau }\right).$$

Process 5: Delay of inhibitory pulse.

$$N_{\Lambda }\left(t\right)=N\left(t-\Lambda_{\text{noi}}\right).$$

Process 6: Gating.

$$C\left(t\right)=I\left(t\right)\left(1-N_{\Lambda }\left(t\right)\right).$$

Process 7: Delay of plasticity trace.

$$\Pi \left(t\right)=H\left(T\left(t-\Lambda_{\text{noi}}\right)\right).$$

Process 8: Coincidence detection.

$$w\left(t\right)=w\left(t-s\right)+\Pi \left(t\right)\left(\delta_{p}-C\left(t\right)\delta_{d}\right).$$

### 2 Derivation of the update function of the adaptive calibration model

We assume that during spontaneous activity, detections in the IO and the PN are independent. Consequently, the probability of a simultaneous detection in both channels is defined as PN_far_IO_far_.

(A1) $${\bar{D}}_{3}=\text{P}{\text{N}}_{\text{far}}\Lambda_{\tau }\text{I}{\text{O}}_{\text{far}}T_{3},$$

where *T*_3_ is the duration of the original recording used for the calibration and Λ*_τ_* is the duration of the plasticity trace.

The linear system to optimize is given by:

(A2) $$\left[\begin{array}{cc} {\bar{P}}_{1} & {\bar{D}}_{1}\\ {\bar{P}}_{2} & {\bar{D}}_{2}\\ {\bar{P}}_{3} & {\bar{D}}_{3} \end{array}\right]\left[\begin{array}{c} \delta_{p}\\ -\delta_{d} \end{array}\right]=\left[\begin{array}{c} -\Delta_{a}∕T_{a}\\ \Delta_{e}∕T_{e}\\ 0 \end{array}\right].$$

The expression in matrix notation becomes:

(A3) A x=b.

Adding the cost matrices for the weighted least squares, we get:

C A x=C b,

where

(A4) $$C=\left[\begin{array}{ccc} c_{1} & 0 & 0\\ 0 & c_{2} & 0\\ 0 & 0 & c_{3} \end{array}\right].$$

With this, the least squares solution for **x** is given by

$$x={\left(A^{T}\mathrm{CA}\right)}^{-1}A^{T}C\;b.$$

First, **A***^T^***CA** expressed in terms of plasticity events and costs is the following matrix

(A5) $$\left[\begin{array}{cc} c_{1}{\bar{P}}_{1}^{2}+c_{2}{\bar{P}}_{2}^{2}+c_{3}{\bar{P}}_{3}^{2} & c_{1}{\bar{P}}_{1}{\bar{D}}_{1}+c_{2}{\bar{P}}_{2}{\bar{D}}_{2}+c_{3}{\bar{P}}_{3}{\bar{D}}_{3}\\ c_{1}{\bar{P}}_{1}{\bar{D}}_{1}+c_{2}{\bar{P}}_{2}{\bar{D}}_{2}+c_{3}{\bar{P}}_{3}{\bar{D}}_{3} & c_{1}{\bar{D}}_{1}^{2}+c_{2}{\bar{D}}_{2}^{2}+c_{3}{\bar{D}}_{3}^{2} \end{array}\right].$$

Grouping and renaming all the terms that do not depend on ${\bar{D}}_{3}$, we can express this matrix as:

(A6) $$\left[\begin{array}{cc} \chi_{1} & \chi_{2}+\psi_{2}{\bar{D}}_{3}\\ \chi_{2}+\psi_{2}{\bar{D}}_{3} & \chi_{3}+\psi_{3}{\bar{D}}_{3}^{2} \end{array}\right].$$

The expressions for the *χ* and ψ terms are given at the end of this Appendix.

The determinant of this matrix, 𝔇, expressed as polynomial of ${\bar{D}}_{3}$ is

$$\left(\chi_{1}\psi_{3}+\psi_{2}^{2}\right){\bar{D}}_{3}^{2}+2\chi_{2}\psi_{2}{\bar{D}}_{3}+\left(\chi_{1}\chi_{3}-\chi_{2}^{2}\right).$$

Using Equation (A1), we can simplify the notation, and express this determinant as a polynomial of IO_far_, which is the input variable that will be updated at each recalibration.

$$\beta_{2}{\text{IO}}_{\text{far}}^{2}+\beta_{1}\text{I}{\text{O}}_{\text{far}}+\beta_{0}.$$

Now we can express (**A***^T^***CA**)^−1^**A***^T^***C** as

$$\frac{1}{𝔇}\left[\begin{array}{cc} \chi_{3}+\psi_{3}{\bar{D}}_{3}^{2} & -\chi_{2}-\psi_{2}{\bar{D}}_{3}\\ -\chi_{2}-\psi_{2}{\bar{D}}_{3} & \chi_{1} \end{array}\right]\left[\begin{array}{ccc} c_{1}{\bar{P}}_{1} & c_{2}{\bar{P}}_{2} & c_{3}{\bar{P}}_{3}\\ c_{1}{\bar{D}}_{1} & c_{2}{\bar{D}}_{2} & c_{3}{\bar{D}}_{3} \end{array}\right],$$

where the first two factor are the inverse of **A***^T^***CA** and the third term in the result of **A***^T^***C**.

Performing the algebra, we obtain the following matrix expression:

$$\left[\begin{array}{cc} \begin{array}{c} \left(\chi_{3}+\psi_{3}{\bar{D}}_{3}^{2}\right)c_{1}{\bar{P}}_{1}\\ \;-\left(\chi_{2}+\psi_{2}{\bar{D}}_{3}\right)c_{1}{\bar{D}}_{1} \end{array} & \begin{array}{c} \left(\chi_{3}+\psi_{3}{\bar{D}}_{3}^{2}\right)c_{2}{\bar{P}}_{2}\\ \;-\left(\chi_{2}+\psi_{2}{\bar{D}}_{3}\right)c_{2}{\bar{D}}_{2} \end{array}\\ \chi_{1}c_{1}{\bar{D}}_{1}-\left(\chi_{2}+\psi_{2}{\bar{D}}_{3}\right)c_{1}{\bar{P}}_{1} & \chi_{1}c_{2}{\bar{D}}_{2}-\left(\chi_{2}+\psi_{2}{\bar{D}}_{3}\right)c_{2}{\bar{P}}_{2} \end{array}\right],$$

where we have omitted the third column of the matrix, since it will be canceled by the zero in the third of row **b**.

The last step to solve the system is to multiply by *b*, after what we obtain the solution for the *δ_p_* and *δ_p_*:

$$\begin{array}{llll} \delta_{p} & =-\frac{1}{𝔇}\frac{\Delta_{a}}{T_{a}}\left(\left(\chi_{3}+\psi_{3}{\bar{D}}_{3}^{2}\right)c_{1}{\bar{P}}_{1}-\left(\chi_{2}+\psi_{2}{\bar{D}}_{3}\right)c_{1}{\bar{D}}_{1}\right)\; & & \;\\ & \;+\frac{1}{𝔇}\frac{\Delta_{e}}{T_{e}}\left(\left(\chi_{3}+\psi_{3}{\bar{D}}_{3}^{2}\right)c_{2}{\bar{P}}_{2}-\left(\chi_{2}+\psi_{2}{\bar{D}}_{3}\right)c_{2}{\bar{D}}_{2}\right)\; & & \; \end{array}$$

and

$$\begin{array}{llll} \delta_{d} & =\frac{1}{𝔇}\frac{\Delta_{a}}{T_{a}}\left(\chi_{1}c_{1}{\bar{D}}_{1}-\left(\chi_{2}+\psi_{2}{\bar{D}}_{3}\right)c_{1}{\bar{P}}_{1}\right)\; & & \;\\ & \;-\frac{1}{𝔇}\frac{\Delta_{e}}{T_{e}}\left(\chi_{1}c_{2}{\bar{D}}_{2}-\left(\chi_{2}+\psi_{2}{\bar{D}}_{3}\right)c_{2}{\bar{P}}_{2}\right),\; & & \; \end{array}$$

where we see that ${\bar{D}}_{3}$ appears in the numerator of *δ_p_* with degree 2 and in the numerator of *δ_d_* with degree 1. In both cases, the denominator is given by the determinant 𝔇, which is another polynomial of degree 2. Therefore, we can express each parameter as a ratio of two polynomials.

$$\begin{array}{llll} & \delta_{p}=\frac{\alpha_{2}{\text{IO}}_{\text{far}}^{2}+\alpha_{1}\text{I}{\text{O}}_{\text{far}}+\alpha_{0}}{\beta_{2}{\text{IO}}_{\text{far}}^{2}+\beta_{1}\text{I}{\text{O}}_{\text{far}}+\beta_{0}},\; & & \;\\ & \delta_{p}=\frac{\gamma_{1}\text{I}{\text{O}}_{\text{far}}+\gamma_{0}}{\beta_{2}{\text{IO}}_{\text{far}}^{2}+\beta_{1}\text{I}{\text{O}}_{\text{far}}+\beta_{0}}.\; & & \; \end{array}$$

In consequence, the adaptive calibration algorithm requires to pre-compute 8 coefficients. The expression for each coefficient is detailed next:

$$\begin{array}{llll} & \begin{array}{c} \beta_{0}=\left(\chi_{1}\chi_{3}-\chi_{2}^{2}\right) \end{array},\; & & \;\\ & \beta_{1}=2\chi_{2}\psi_{2},\; & & \;\\ & \beta_{2}=\left(\chi_{1}\psi_{3}+\psi_{2}^{2}\right),\; & & \;\\ & \alpha_{0}=\frac{\Delta_{a}}{T_{a}}\left(\chi_{2}c_{1}{\bar{D}}_{1}-\chi_{3}c_{1}{\bar{P}}_{1}\right)+\frac{\Delta_{e}}{T_{e}}\left(\chi_{3}c_{1}{\bar{P}}_{1}-\chi_{2}c_{2}{\bar{D}}_{2}\right),\; & & \;\\ & \alpha_{1}=\frac{\Delta_{a}}{T_{a}}\psi_{2}c_{1}{\bar{D}}_{1}-\frac{\Delta_{e}}{T_{e}}\psi_{3}c_{2}{\bar{D}}_{2},\; & & \;\\ & \alpha_{2}=\frac{\Delta_{e}}{T_{e}}\psi_{3}c_{2}{\bar{P}}_{2}-\frac{\Delta_{a}}{T_{a}}\psi_{3}c_{1}{\bar{P}}_{1},\; & & \;\\ & \gamma_{0}=\frac{\Delta_{a}}{T_{a}}\left(\chi_{1}c_{1}{\bar{D}}_{1}-\chi_{2}c_{1}{\bar{P}}_{1}\right)+\frac{\Delta_{e}}{T_{e}}\left(\chi_{1}c_{2}{\bar{D}}_{2}-\chi_{2}c_{2}{\bar{P}}_{2}\right),\; & & \;\\ & \gamma_{1}=\frac{\Delta_{e}}{T_{e}}\psi_{2}c_{2}{\bar{P}}_{2}-\frac{\Delta_{a}}{T_{a}}\psi_{2}c_{1}{\bar{P}}_{1},\; & & \; \end{array}$$

where *χ* and ψ are given by

$$\begin{array}{llll} & \chi_{1}=c_{1}{\bar{P}}_{1}^{2}+c_{2}{\bar{P}}_{2}^{2}+c_{3}{\bar{P}}_{3}^{2},\; & & \;\\ & \chi_{2}=c_{1}{\bar{P}}_{1}{\bar{D}}_{1}+c_{2}{\bar{P}}_{2}{\bar{D}}_{2},\; & & \;\\ & \chi_{3}=c_{1}{\bar{D}}_{1}^{2}+c_{2}{\bar{D}}_{2}^{2},\; & & \;\\ & \psi_{2}=c_{3}{\bar{P}}_{3},\; & & \;\\ & \psi_{3}=c_{3}.\; & & \; \end{array}$$
